# The High‐Altitude Adaptation Characteristics of Microbiota‐Host Cross‐Talk in Yak Gastrointestinal Track

**DOI:** 10.1002/advs.202514862

**Published:** 2025-10-27

**Authors:** Chun Huang, Minghao Zhang, Qingbo Zheng, Qinran Yu, Guowu Yang, Wenwen Ren, Xiaoming Ma, Yongfu La, Pengjia Bao, Min Chu, Xian Guo, Chunnian Liang, Ping Yan

**Affiliations:** ^1^ Lanzhou Institute of Husbandry and Pharmaceutical Sciences Chinese Academy of Agricultural Sciences (Key Laboratory of Animal Genetics and Breeding on Tibetan Plateau, Ministry of Agriculture and Rural Affairs, Key Laboratory of Yak Breeding Engineering of Gansu Province) Lanzhou 730050 China; ^2^ Institute of Western Agriculture Chinese Academy of Agricultural Sciences Changji 831100 China

**Keywords:** cross‐species analysis, hypoxia, microbiota‐host crosstalk, multi‐tissue single‐cell transcriptomic atlas, yak gastrointestinal tract

## Abstract

The yak, an ideal model for studying high‐altitude hypoxia adaptation, possesses unique gastrointestinal tract (GIT) adaptability. However, understanding of cellular‐level mechanisms underlying host‐metabolite‐microbe within GIT that are crucial for growth in extreme environments remains significantly limited. Therefore, this study constructs the first comprehensive multi‐tissue cellular atlas of the yak GIT, encompassing 54 distinct cell types. Cross‐species and cross‐tissue comparative analyses combined with large‐scale population genetic data identify *HNF4A* and *SREBF2* as GIT‐specific transcription factors targeting the key gene *MYO6*, revealing unique transcriptional patterns and the significant influence of epithelial cells on yak body weight in GIT. Alongside the characterization of microorganisms and metabolites along the GIT, the important microorganism *Bacillus* infection has cell‐type specificity, and affects the accumulation of key products such as Succinate and lactic acid through the interaction between different epithelial cell metabolic activities and microorganisms and the communication between different cell types (key receptors *SLC27A5*, *PPARA*), thereby affecting glycolysis and TCA cycle and other processes to strengthen the adaptability of yak GIT in extreme environments. This work provides novel insights into the unique gastrointestinal adaptations of yaks to extreme environments and holds significant implications for understanding precision breeding in yaks and mammal gastrointestinal responses to hypoxia.

## Graphical abstract

In this study, a single‐cell atlas of 117,019 yak gastrointestinal cells across 54 subtypes identified *HNF4A* and *SREBF2* as key transcription factors targeting *MYO6* gene. Cross‐species and multi‐omics analyses reveals epithelial cells as key regulators that, through interactions with microbes, particularly *Bacillus*, facilitate flexible energy supply and glycogen storage to cope with the challenges of yak growth under high‐altitude conditions.

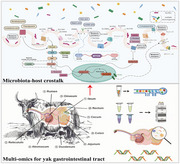

## 1 Introduction

The yak (*Bos grunniens*) is a large ruminant capable of efficiently exploiting alpine grassland resources. In response to the extreme environmental conditions of the plateau, including hypoxia, severe cold, intense ultraviolet radiation (UVR), and food scarcity, yaks have developed stable and distinctive genetic adaptations. This makes them an ideal model for investigating mammalian adaptation to high altitudes. The grazing activities of yaks not only facilitate the dispersal of plant seeds and microorganisms but also play a vital role in material cycling within the ecosystem,^[^ ^1^ ^]^ serving as a key factor in maintaining ecological balance and biodiversity on the Qinghai‐Tibet Plateau (QTP). Yaks were domesticated approximately 7300 years ago and possess the remarkable ability to convert indigestible fibrous forage into essential human foods, such as meat and dairy products.^[^ ^2^ ^]^ It has been reported that nearly 582 million people will be at risk of malnutrition by 2030.^[^ ^3^ ^]^ Therefore, to meet the increasing demand for healthy and environmentally friendly food while minimizing ecological impact, it is essential to understand and explore the mechanisms underlying yak adaptation to high‐altitude environments. In particular, the gastrointestinal regulatory mechanisms involved in nutrient absorption and energy metabolism are closely associated with economically important traits and play a critical role in enhancing food production.

The gastrointestinal tract (GIT) plays a pivotal role in the adaptability of high‐altitude animals and is regarded as a critical link in converting forage nutrients into host‐available energy. Studies on Tibetan chickens,^[^ ^4^ ^]^ Tibetan sheep,^[^ ^5^ ^]^ and Tibetan pigs^[^ ^6^ ^]^ have demonstrated that the GIT of high‐altitude animals exhibits unique adaptations in morphological structure, digestive enzyme activity, and microbial composition. Notably, short‐chain fatty acids (SCFAs) produced in the GIT not only improve energy utilization efficiency but also reduce methane emissions through microbial regulation, thereby enhancing survival capacity under extreme environmental conditions. However, current research on the yak GIT has primarily relied on bulk RNA sequencing, including studies of the four‐chambered (FC) stomach^[^ ^7^ ^]^ and intestine,^[^ ^8^ ^]^ as well as investigations into how factors such as season, diet, feeding systems, and age influence its microbiota and metabolites.^[^ ^9^, ^10^ ^]^ While these studies have provided insights into gene expression dynamics and identified key functional genes, they overlook the intrinsic heterogeneity of gene expression at the single‐cell level and the dynamic interactions among host, metabolites, and microorganisms across distinct GIT regions. These interactions regulate diverse digestive, immune, and endocrine processes, which are essential for improving animal production performance.^[^ ^11^, ^12^ ^]^ Therefore, constructing a multi‐tissue single‐cell atlas of the yak GIT, systematically exploring the mechanisms by which specific cell types regulate complex traits, and elucidating the intricate relationships between host metabolites and the microbiome are of great significance for optimizing gastrointestinal function and deepening our understanding of high‐altitude adaptation.

**Figure 1:**
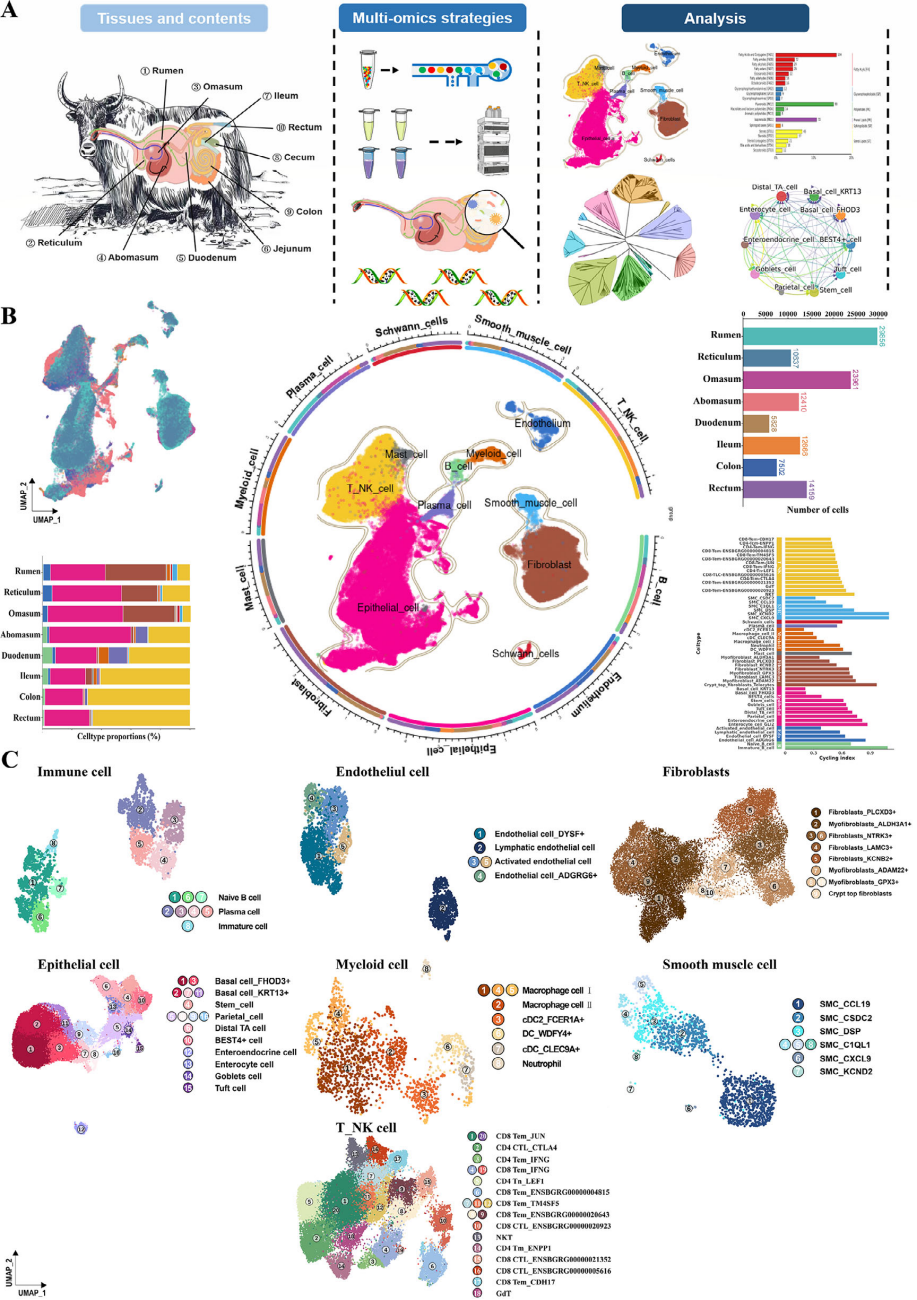
Single‐cell transcriptomic landscape from yak gastrointestinal tract (GIT). A) A schematic diagram of tissues and an overview of the multi‐omics study design and bioinformatics analyses. B) UMAP visualization of a total of 117 019 high‐quality cells from yak GIT tissues by major cell types. Top‐left: The global cell types of all cells from the dataset colored by tissue with UMAP visualization. Bottom‐left: The proportion of each major cell type in different tissues. Top‐right: The bar plots of the number of high‐quality cells for each tissue. Bottom‐right: The bar chart of cell cycling index for each cell type. C) UMAP hvisualization of cell clusters and cell‐type annotation for each cluster is provided in the right panel.

In light of the above considerations, we construct the first single‐cell transcriptomic atlas of gastrointestinal tissues in adult yaks. By integrating this atlas with genome‐wide association study (GWAS) data from 247 yaks and cross‐species scRNA profiles, we identify the key transcription factors (*HNF4A* and *SREBF2*) regulating *MYO6*, with implications for nutrient absorption and energy metabolism in the high‐altitude environment (**Figure** **1**A). Furthermore, we profile the distribution characteristics of *Bacillus* within the cells and highlight that epithelial cells engage in cross‐talk with microorganisms and metabolites by regulating energy storage strategies in yaks to cope with the challenges posed by the high‐altitude hypoxic environment. Collectively, this study augments resources for yak‐related research, offering new perspectives on the mechanisms of adaptation to high‐altitude environments and investigations into plateau‐associated diseases in yaks.

## 2 Results

### 2.1 Global Landscape of Single‐Cell Transcriptomic Reference Atlas from Yak Gastrointestinal Tract

We constructed a multi‐tissue single‐cell transcriptomic reference atlas of the yak GIT, which includes the FC stomach (rumen, reticulum, omasum, abomasum), small intestine (duodenum and ileum), and large intestine (colon and rectum). Each sample generated over 400 million reads on average, and after stringent quality control, a total of 117 019 high‐quality cells were retained for subsequent analyses. The rumen contributed the largest number of cells (*n* = 29 856), whereas the duodenum yielded the fewest 5828 cells (Figure 1B). Ten major cell types were identified based on three distinct methods, predominantly including epithelial cells, fibroblasts, endothelium cells, and others (see Experimental Section) (Figure 1B, Table S3, Supporting Information). The tissue‐specific distribution of major cell types revealed organ‐level transcriptional specificity across different tissues, whereas tissues of the same organ showed comparable cellular compositions (Figure S1, Supporting Information). In the FC stomach, fibroblasts and epithelial cells predominated, while a progressive decline in fibroblasts and a marked increase in T and NK (T_NK) cells were observed along the GIT. These shifts likely reflected functional specialization across digestive organs (Figure 1B), suggesting that yaks may promote efficient nutrient utilization and maintain mucosal homeostasis by enhancing local immune defense, which could be crucial for their ability to effectively process high‐fiber diets under high‐altitude conditions. Subsequently, we identified 54 distinct cell subtypes in GIT (Figure 1C). Each subtype contained sufficient numbers of UMIs and detected genes to ensure reliable classification. Notably, we detected BEST4⁺ cells in yaks, consistent with findings in humans,^[^ ^13^ ^]^ mice,^[^ ^14^ ^]^ pigs,^[^ ^15^ ^]^ rhesus macaques,^[^ ^15^ ^]^ and rabbits.^[^ ^16^ ^]^ These BEST4⁺ cells represent a novel subtype of mature absorptive epithelial cells involved in pH regulation and electrolyte secretion,^[^ ^17^, ^18^ ^]^ potentially contributing to the maintenance of gastrointestinal acid–base balance and facilitating adaptation to the unique physiological challenges posed by the plateau environment in yaks. This cell type has not been reported in other ruminants such as cows^[^ ^19^ ^]^ or donkeys,^[^ ^20^ ^]^ where Paneth‐like cells, morphologically and transcriptionally similar to BEST4⁺ cells, were identified instead. To investigate cell‐cycle status, we calculated the cycling index for each cell type (Figure 1B). Immune‐related cells, including B cells and T_NK cells, exhibited high cycling indices, indicative of active proliferation. In contrast, myeloid and endothelial cells were largely quiescent (Figure 1B).

### 2.2 Tissue‐Specific Expression Patterns in the Yak GIT

**Figure 2:**
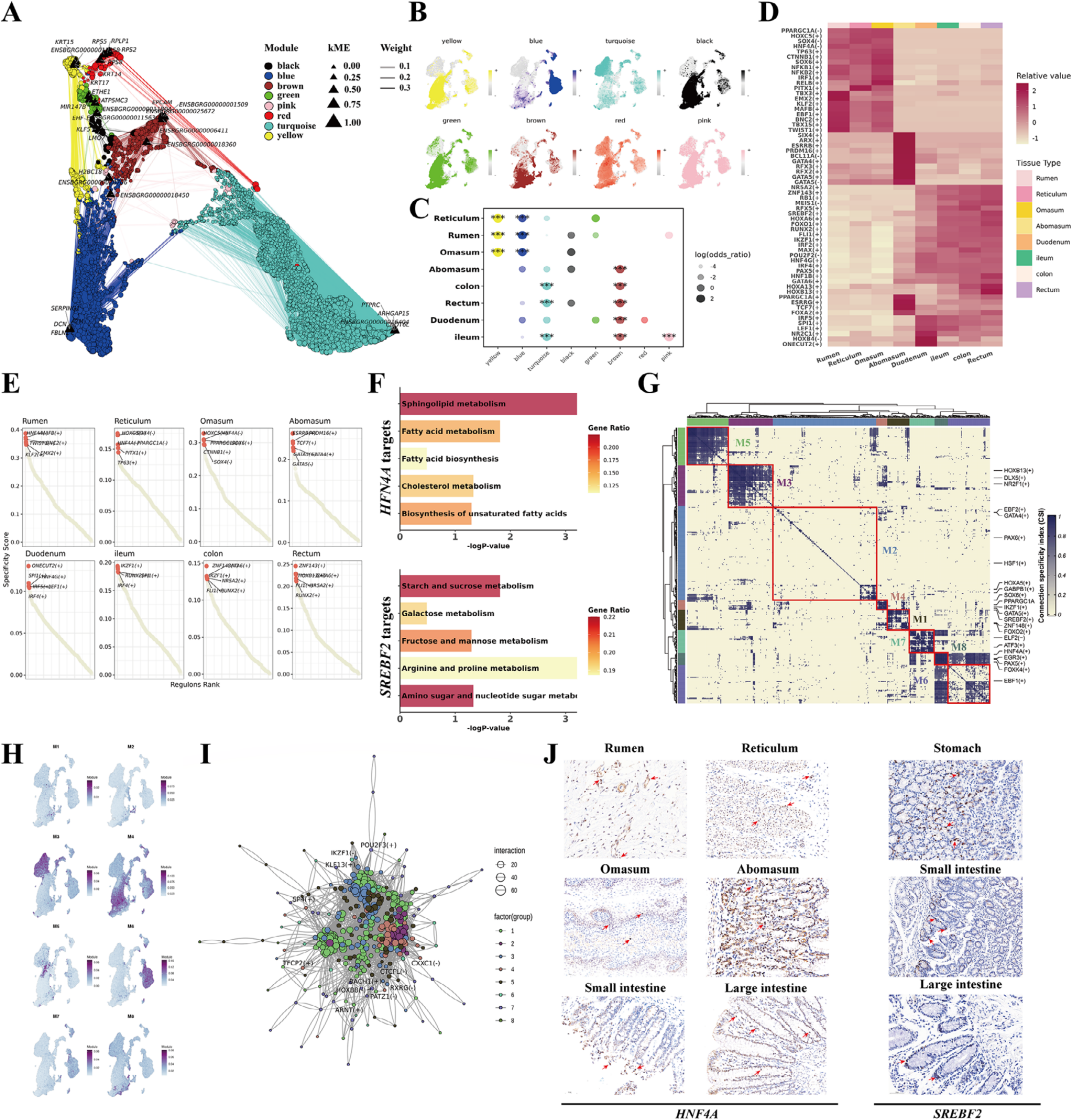
Characterization of molecule and transcription factors across tissues. A) TOM UMAP plot of the yak tissues co‐expression network. Each node represents a single isoform, and edges represent co‐expression links between isoforms and module hub isoforms. Nodes are colored by co‐expression module assignment. B) UMAP visualization of expression patterns of module eigengenes. C) Co‐expression modules were compared with marker genes of yak tissue. The size of the dots represents the number of overlapping genes. FDR significance levels as stars on top of the dots. “***”: 0−0.001; “**”: 0.001−0.01; “*”: 0.01−0.05; (No symbol): >0.05. D) Heatmap illustrating the specific TFs analysis of different tissues based on the RSSZ. E) The specificity ranking plot of regulons in each tissue, the abscissa represents the ranking, and the ordinate represents the RSS specificity score. The higher RSS regulator may be related to the tissue specificity. Orange point represents the top six TFs with RSS score. F) The KEGG analysis of target genes of *HFN4A* and *SREBF2*. G) Identification of combinatorial regulon modules based on regulon CSI matrix. H) UMAP illustrating the average AUCell score distribution for different regulon modules. I) TF–TF network for the cell types based on similarity of regulon activities. J) Representative IHC staining of *HNF4A* and *SREBF2* in yak stomach, small intestine, and large intestine.

To investigate tissue‐specific genes involved in distinct biological functions, we characterized transcriptional profiles across different GIT tissues. Co‐regulatory interaction analysis using hdWGCNA identified eight tissue‐associated gene modules, with the yellow and blue modules enriched in the FC stomach and the pink module enriched in the ileum (**Figure** **2**A–C; Table S4 and Figure S2, Supporting Information). We identified hypoxia‐adaptation‐related genes such as *PPARA*, *VEGFG*, and *MYDGF*, which significantly activated GO terms including blood vessel development, anatomical structure development, and macromolecule biosynthetic regulation (Table S5, Supporting Information). These findings suggest that the yak GIT may enhance nutrient delivery efficiency in coordination with immune regulation, thereby alleviating hypoxia‐ and cold‐induced metabolic and barrier stress, ultimately maximizing digestive absorption and energy metabolism under high‐altitude conditions.

TFs are of paramount importance in regulating gene expression. Sixty‐one TFs were identified across eight tissues based on Z‐score normalized regulon specificity score analysis (Figure 2D, Table S6, Supporting Information). Abomasum had the highest number of active TFs, indicating a unique transcriptional landscape and a potential link between tissue‐specific TFs and cellular identity. In contrast, the FC stomachs and intestine displayed similar transcriptional profiles within their respective groups, implying that certain TFs may function similarly across related tissues. For example, *HNF4A* exhibited high activity in fore‐stomach, whereas *GATA6* and *ZNF143* were more active in the colon and rectum. Ranking tissue‐enriched regulons by RSS scores revealed that fore‐stomach *HNF4A* target genes were involved in energy metabolism (*Acat1*,^[^ ^21^ ^]^ *ACSL5*,^[^ ^22^ ^]^ *PGC‐1α* ^[^ ^23^ ^]^) and nutrient absorption, notably SLC family genes mediating VFA anion exchange (Figure 2E). In the intestine, *SREBF2* targets associated with fatty acid transport (*FABP6*),^[^ ^24^ ^]^ bile acid metabolism (*SLC10A2*),^[^ ^25^ ^]^ and glucose transport (*SLC2A2*).^[^ ^26^ ^]^ KEGG analysis of *HNF4A* and *SREBF2* targets revealed activation of glucose and fatty acid metabolism, and insulin signaling, involving hypoxia‐adaptation genes such as *PPARA* and *AMOT*, which may enhance glycolysis, nutrient delivery, and carbon source flexibility to maintain energy homeostasis and thermogenesis in yaks under hypoxic‐cold conditions (Figure 2F). Meanwhile, these TFs were ubiquitously expressed across tissues (Figure 2J). Gene expression levels were frequently regulated through the concerted action of TFs. We systematically identified regulon modules using the connection specificity index (CSI), which revealed that all regulons clustered into eight primary modules (Figure 2G, Table S7, Supporting Information). Module 3 (M3) was predominantly expressed in intestinal tissues, while M4 was stomach‐specific (Figure 2H). TF‐TF regulatory network analysis (Figure 2I) indicated that TFs from different modules could act cooperatively or tissue specifically, with M1, M3, and M5 showing close interconnections suggesting coordinated regulation across cell populations.

### 2.3 Cross‐Species Analysis Revealed Transcriptional Characteristic of Yak GIT

**Figure 3:**
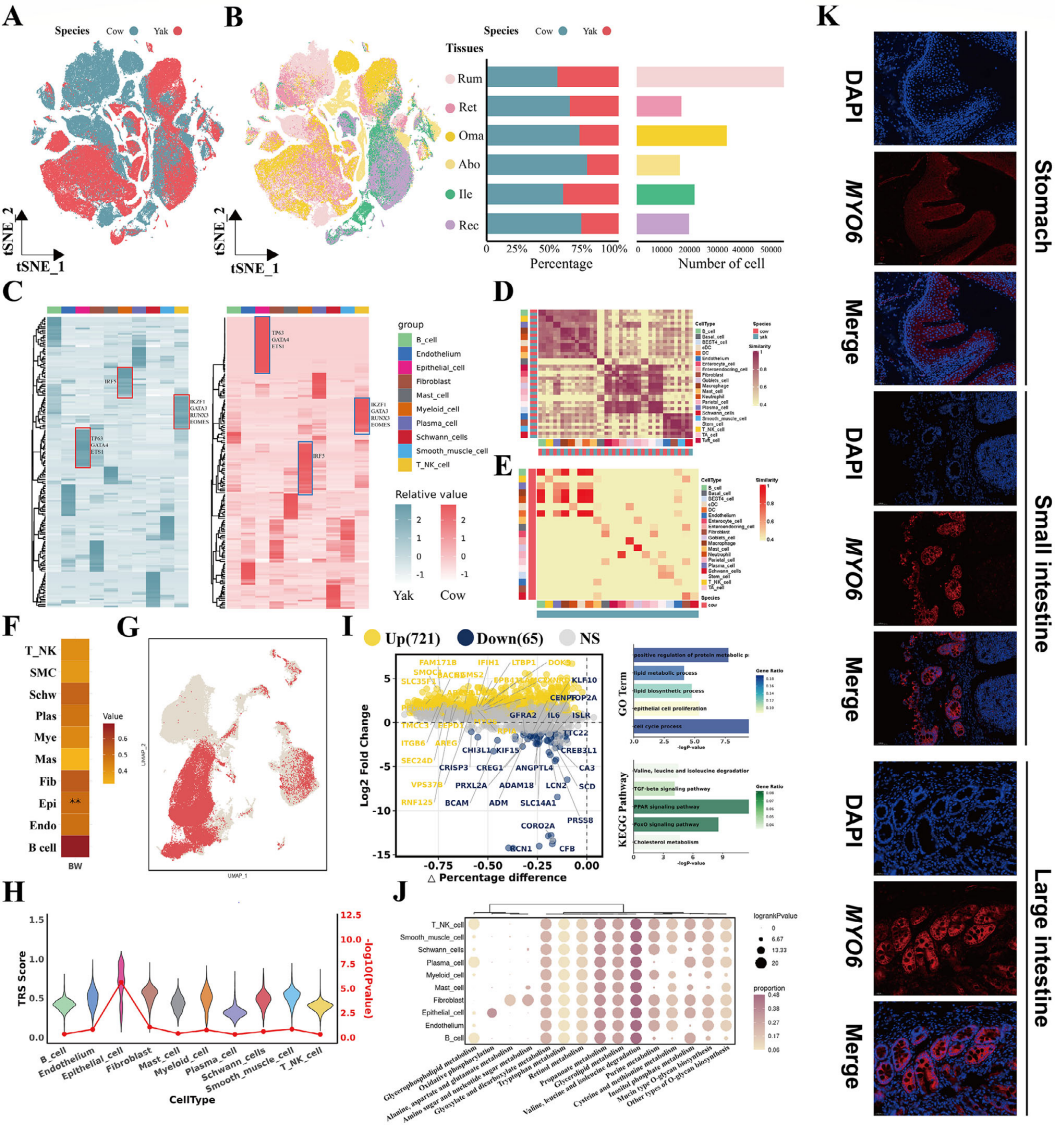
Comparative analysis of cross‐species cellular landscapes. A) A tSNE map of cells colored by species. B) A tSNE map of global cells colored by tissue (left), and bar plots showing the number and percentage of cells for each species (right). C) The heat map shows the transcription factor RSSZ across various cell types in the cow and yak. D) Similarity analysis of orthologous gene expression between cow and yak cell types based on the AUROC scores calculated by MetaNeighbor software. E) The heat map shows the Spearman's correlation of average gene expression between cow and yak. F) The heat map shows the average trait correlation scores (TRSs) for cells belonging to the same cell type, the asterisk represents the association degree between cell type and body weight trait (^∗∗^FDR < 0.01). G) Per‐cell TRSs calculated by scPagwas for body weight trait are shown in UMAP coordinates, red point represents positive cells (*Random_Correct_BG_adjp* <0.05), other point represents negative cells. H) Correlations between cell types and the body weight trait. The violin plot represents the trait‐relevant score (TRS) for each cell type with the corresponding trait. The red line represents the significant level (−log_10_ *^p^* ^‐value^) for each cell type with the corresponding trait. I) The volcano plot displaying differentially expressed genes (DEGs) of epithelial cells between the cow and yak (left). GO and KEGG enrichment analysis of upregulated genes in epithelial cells of yak (right). J) Dot plot demonstrating the body weight‐relevant pathways in different cell types identified by scPagwas, and color intensity indicates the proportion of cells within each cell type genetically influenced by a given pathway (pathway‐level coefficient *β* > 0). K) Representative images of *MYO6* staining (red) in different tissues of yak. Nucleus was counterstained with DAPI (blue).

Cross‐species analysis is critical for identifying yak‐specific gastrointestinal molecular features and elucidating its unique mechanisms of nutrient absorption and metabolism. The cow is a well‐characterized ruminant and serves as an excellent low‐altitude mammalian model for comparative studies of plateau adaptation alongside the yak. The t‐distributed stochastic neighbor embedding (t‐SNE) of 164 573 high‐quality revealed cell characteristics across species and tissues, with cell numbers per bovine tissue ranging from 3908 in the abomasum to 25 825 in the rumen (**Figure** **3**A,B). Shared cell‐type annotations revealed that the expression patterns of representative marker genes were largely conserved between yak and cow. We identified 165 and 157 cell‐type specific TFs in yak and cow, respectively, among which 38 were shared across corresponding cell types (Figure 3C). Several TFs exhibited conserved regulatory activity, exemplified by *GATA4* and *ETS1* in epithelial cells and *EOMES* in T_NK cells, highlighting conserved transcriptional regulation in major gastrointestinal cell types of both species.

The similarity analysis using MetaNeighbor revealed that immune and myeloid cells displayed highly conserved gene expression between yak and cow, whereas epithelial cells and fibroblasts showed lower correlation, likely reflecting terminal differentiation states and species‐specific functions (Figure 3E). Integrating whole‐genome sequencing and GWAS summary statistics from 247 yaks using scPagwas identified epithelial cells as the only cell type significantly associated with body weight (*p* <Z0.01, Figure 3F). At the single‐cell level, epithelial cells contained the highest proportion of positive cells based on trait correlation scores (TRS), consistent with previous findings in pigs that linked production traits to epithelial cells^[^ ^27^ ^]^ (Figure 3G,H), suggesting that epithelial cells make significant contributions to the growth of yaks in high‐altitude environments. Furthermore, epithelial cells displayed high TRS which underscoring the critical roles of these cell types in regulating the production performance of yaks (Figure 3H). Specific genetic pathway activities pathway analysis further showed significant enrichment in glycerophospholipid metabolism, Thermogenesis, and Hedgehog signaling (Figure 3J). Together, these results highlight epithelial cells as the central regulators of yak production traits, with enhanced metabolic flexibility and proliferative capacity that likely underpin efficient nutrient absorption and sustained energy supply for adaptation to high‐altitude hypoxic environments.

Furthermore, differentially expressed genes (DEGs) between yak and cow epithelial cells elucidated the yak‐specific characteristics (Figure 3I). A total of 721 genes were upregulated and 65 downregulated in yak epithelial cells. Upregulated genes, including *SLC35F1*, *MYO6*, and *PECAM1*, were enriched in protein regulation, lipid metabolism, and epithelial proliferation, as well as pathways related to amino acid degradation and *FoxO* signaling, suggesting enhanced nutrient absorption and metabolic efficiency. Notably, *MYO6*, a target of yak‐specific TFs *HNF4A* and *SREBF2*, showed markedly higher expression in epithelial cells and was validated by IF staining as a specific epithelial marker (Figure 3K). These findings underscore epithelial cell–intrinsic transcriptional programs that may facilitate efficient energy utilization and contribute to yak adaptation to hypoxia and cold at high altitude.

### 2.4 Profiling of Microbiome Composition, Taxonomy, and Function in Yak Gastrointestinal Tract

**Figure 4:**
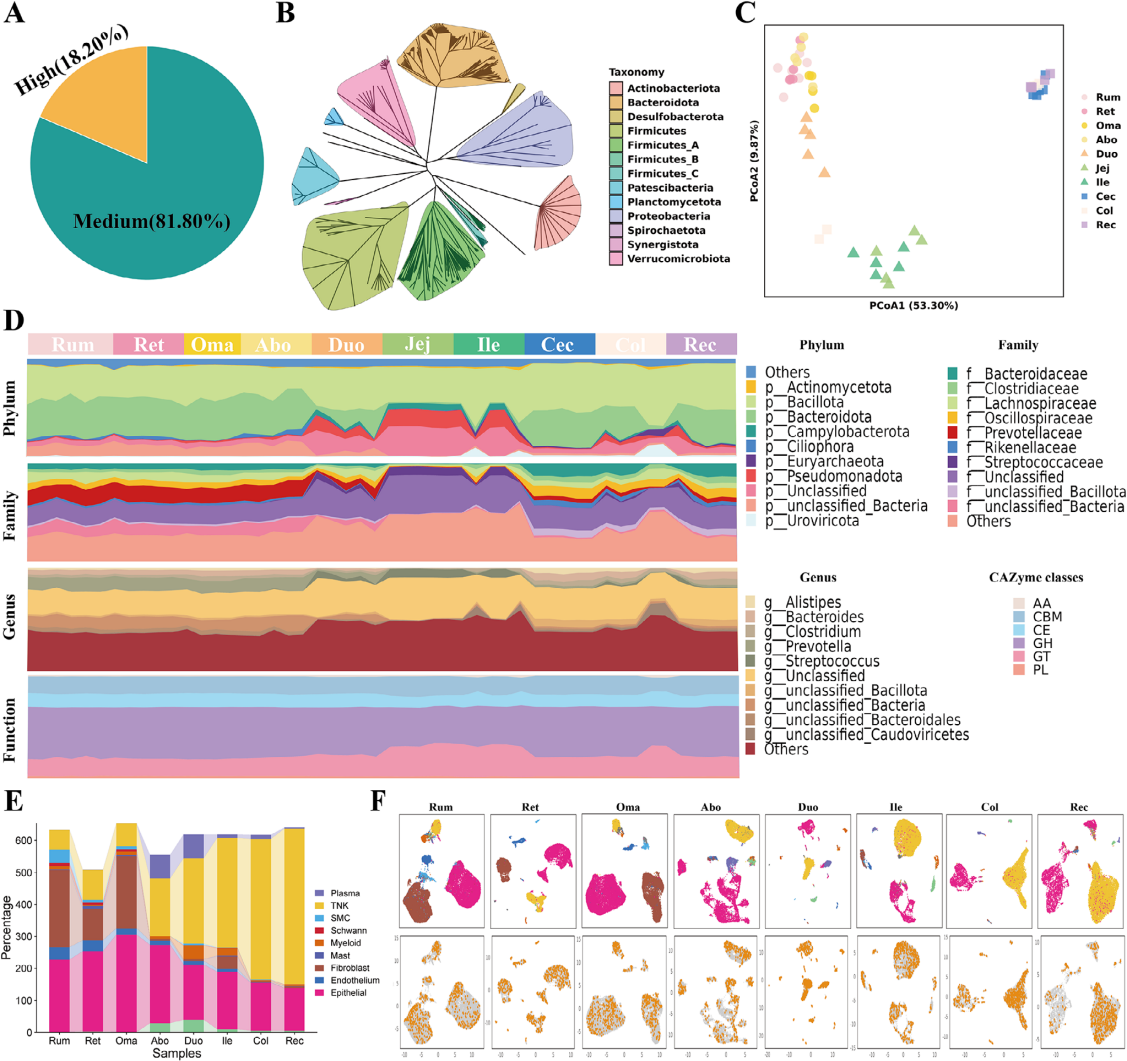
Overview of microorganism characteristics along the yak gastrointestinal tract (GIT) site based on MAGs. A) The pie chart shows the number and relative proportion of MAGs for subsequent analysis. B) Taxonomic composition of 533 GIT MAGs at the phylum level. Phyla are highlighted with different background colors. C) PCoA plot based on Bray–Curtis distance of microorganism abundance of different tissues. D) Stream graph shows the detailed relative abundances of top phylum, family genus, and CAZyme class along the yak GIT. The *X*‐axis indicates the samples clustered by the sampling sites along the GIT. The *Y*‐axis indicates the relative abundances in each sample. E) The bar plot shows the number of Bacillus‐infected cells in different cell types of each tissue. F) UMAP shows the distributions of *Bacillus*‐infected cells. The cell landscape of different tissues is shown above, and the distribution of *Bacillus*‐infected cells in the corresponding tissues is shown below. The orange point represents the *Bacillus*‐infected cell, and the gray point represents the *Bacillus*‐uninfected cell.

The unique gastrointestinal structure of ruminants fundamentally distinguishes food processing and nutrient utilization from those of nonruminant animals. Different regions of GIT perform specialized functions that be often tightly coupled with the composition and metabolic activity of region‐specific microbial communities. We employed metagenome assembly and binning approaches to characterize the microbial diversity and dynamics along the yak GIT and individual microbial genomes potentially involved in nutrient utilization. 3 151 142 462 clean reads were retained across all samples, with an average of 63 022 849 clean reads and 9392532065 clean bases per sample (Table S8, Supporting Information). We obtained 128061969 contigs, which were subsequently grouped into 10759 bins. After filtering based on genome completeness (≥70%) and contamination (≤10%), a nonredundant set of 533 MAGs was retained for downstream analysis (Table S10, Supporting Information). A total of 128 MAGs were classified as near‐complete (completeness ≥90%), and 405 MAGs were substantially complete (completeness between 70% and 90%) following the standards defined by Parks et al.^[^ ^28^ ^]^ Among all MAGs, 8 MAGs reached 100% completeness and 368 exhibited low contamination (<5%) (**Figure** **4**A). In general, 521 MAGs were assigned to bacteria with *order* level at least, including 2 *kingdoms*, 18 *phyla*, 23 *classes*, and 41 *orders*, respectively (Table S11, Supporting Information, Figure 4B). However, many MAGs remained unclassified at the *species* level, and 150 MAGs were unassigned even at the *genus* level. At the *phylum* level, *Bacteroidota* was the most dominant group (*n* = 247), followed by *Firmicutes_A* (*n* = 129), *Firmicutes* (*n* = 29), and *Verrucomicrobiota* (*n* = 19). All members of *Bacteroidota* belonged to the class *Bacteroidia*, whereas members of *Firmicutes_A* were classified into *Clostridia* (*n* = 122) and *Clostridia_A* (*n* = 7).

We further elucidated the distribution and compositional variation of microbial communities along GIT, and PCoA results revealed a clear separation among the FC stomach, SI, and LI, with the LI exhibiting the most pronounced divergence. The duodenum exhibited intermediate microbial similarity to fore‐stomachs and SI (Figure 4C). Based on the Shannon index, microbial diversity was highest in the FC and lowest in the SI (Figure S3, Supporting Information). *Firmicutes* (also referred to as *Bacillota*) and *Bacteroidota* were identified as the two dominant *phyla* across the GIT (Figure 4D). Notably, the relative abundance of *Bacillota* was relatively stable along GIT sites, with an increasing trend. *Bacteroidota* displayed a marked decline in SI and a subsequent increase in LI. The ratio of *Firmicutes* to *Bacteroidota* (F/B ratio) has been widely reported as a key indicator of microbial contribution to energy harvest and expenditure.^[^ ^29^ ^]^ In our study, the F/B ratio remained relatively constant in the FC and LI, but fluctuated substantially in the SI, particularly in the jejunum and ileum, where the relative abundance of *Bacteroidota* was significantly reduced. This pattern contrasted with findings in buffalo, where the F/B ratio shows a gradual increase along the digestive tract.^[^ ^30^ ^]^ The elevated F/B ratio in the jejunum and ileum highlighted the important role of the yak small intestine in nutrient uptake and energy acquisition, which may enhance the yak's ability to meet the high energy demands required for survival in high‐altitude or cold environments. The degradation of complex plant fibers in ruminants is primarily facilitated by CAZymes. The overall abundance of CAZyme genes was consistent across most GIT regions, marked differences were observed in the SI. Specifically, glycoside hydrolase (GH) encoding genes were significantly reduced in the SI, whereas glycosyltransferase (GT)‐encoding genes were notably enriched, particularly in the jejunum and ileum.

Using scRNA‐seq data across multiple tissues, we identified known microbial infections by SAHMI computational framework. We found that *Bacillus* was consistently among the top five most frequently detected microbes in infected cells across all tissue types. The presence of *Bacillus* was cell‐type specific, predominantly occurring in epithelial cells and T_NK cells. In epithelial cells, the number of *Bacillus*‐infected cells gradually decreased along the GIT, whereas in T_NK cells, the number increased progressively (Figure 4E). The visualization of *Bacillus genus* onto the scRNA landscape genus indicated that *Bacillus* was widely distributed across different tissues, suggesting that, as a member of the phylum *Firmicutes*, it may not only contribute to energy production for the host but also play a potential role in immune regulation within the yak GIT (Figure 4F).

### 2.5 Metabolic Characteristic of Different Tissues

**Figure 5:**
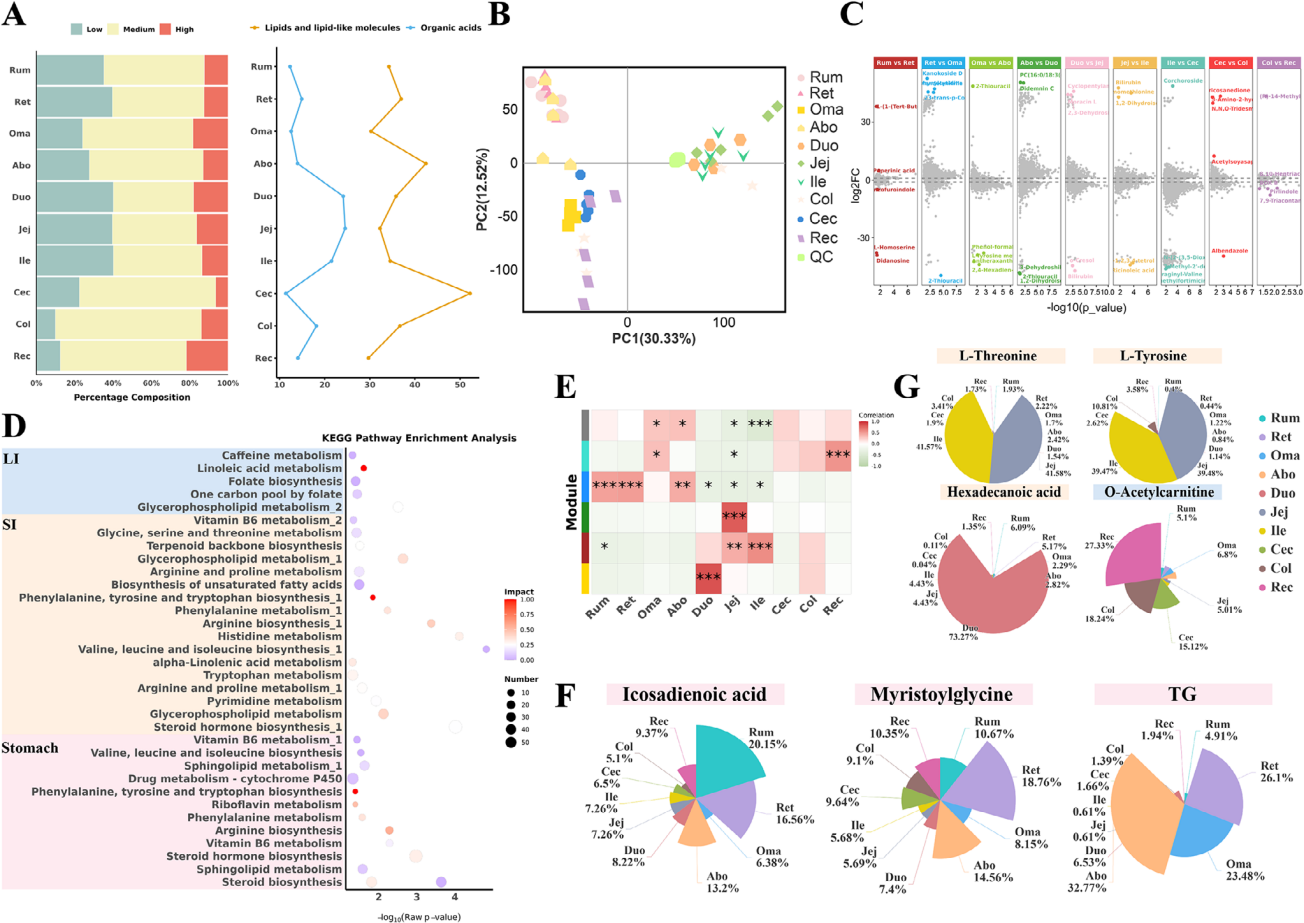
Metabolic characteristic of different tissues in yak gastrointestinal tract (GIT). A) Bar plots based on high, intermediate, and low metabolite levels in the different tissues of yak GIT (left). Line graph showing proportion of metabolite classification in HMDB database (right). B) Principal component analysis (PCA) analysis of metabolome samples from different tissues, points marked with the same color represent the replicates of the same tissues. C) Analysis of differential metabolites along gastrointestinal sites. D) KEGG pathway enrichment analysis of upregulated differential metabolites in different organs. E) Heatmap showing the correlation between different tissues and functional modules identified by WGCNA. F) Relative levels of representative metabolites associated with metabolite categories in the FC stomach, SI and LI. G) The colors of the metabolites are the same as those in Figure D, representing different organs.

We investigated the characteristics of nutrient metabolism across 10 tissues. Metabolites were grouped based on the abundance levels (see Experimental Section; **Figure** **5**A). Tissues from the large intestine exhibited a higher proportion of medium‐abundance metabolites, while those from SI demonstrated more high‐abundance metabolites (Table S12, Supporting Information). The annotation and classification of high‐abundance metabolites using HMDB database indicated that the categories of lipids and lipid‐like molecules, as well as organic acids and derivatives, were the most prevalent among high‐abundance metabolite, especially lipid‐related metabolites (Figure 5A). These results suggested that lipid metabolism in the yak GIT undergoes adaptive adjustments, whereby the enrichment of polyunsaturated fatty acids may provide substrates for energy metabolism and enhance crude protein digestibility to effectively cope with hypoxic challenges. PCA results revealed distinct metabolic signatures across tissues, specifically, tissues within the same organ exhibited significant similarities, whereas the distribution patterns of metabolite categories varied markedly between different organs (Figure 5B). Functional annotation of high‐abundance metabolites in different organs revealed that gastric metabolites were predominantly involved in pathways such as riboflavin metabolism, tyrosine metabolism, and fatty acid synthesis. While intestinal metabolites were primarily associated with amino acid synthesis, TCA cycle, and fatty acid degradation (Table S13, Supporting Information). We identified 97 (Colon versus Rectum) to 2338 (Reticulum versus Omasum) upregulated differential metabolites, and 37 (Colon versus Rectum) to 1458 (Abomasum versus Duodenum) downregulated metabolites across sequential tissues from the rumen to the rectum (Figure 5C). Functional analysis of upregulated metabolites indicated their primary involvement in biosynthesis of lipids, amino acids, and related metabolites in gastric tissues. Upregulated metabolites in intestinal tissues were predominantly enriched in metabolic pathways for amino acid‐related metabolites and the biosynthesis of unsaturated fatty acids, including processes such as linoleic acid metabolism and folate biosynthesis in the large intestine (Figure 5D). Furthermore, we revealed key metabolite co‐expression network by WGCNA. Six distinct modules were identified, among which the blue module showed a significant association with yak stomach tissues (Figure 5E). Based on the module eigengene, metabolite significance of the blue module, and differential metabolites, we screened for key metabolites within yak stomach tissues. Among these, icosadienoic acid, a polyunsaturated fatty acid, was found to be abundant in the stomach, particularly in the rumen (Figure 5F). Regarding protein synthesis, amino acid metabolites such as l‐threonine and l‐tyrosine were also present at higher levels in the small intestine (Figure 5G). These findings indicated that yak gastrointestinal tissues exhibit both functional similarities and distinctions. These metabolic patterns may reflect key mechanisms underlying yak adaptation to high‐altitude environments. Specifically, the enrichment of unsaturated fatty acids in gastric tissues not only provides an efficient energy source under limited oxygen availability but also supports microbial protein synthesis, which is essential for maintaining energy balance in hypoxic and cold conditions. Likewise, the enhanced capacity for amino acid metabolism and vitamin biosynthesis in the small intestine supports greater nutrient absorption and metabolic flexibility, ensuring adequate energy supply and cellular homeostasis under chronic hypobaric hypoxia in yaks.

### 2.6 Metabolic Landscape of Epithelial Cell Types in Different Tissues

**Figure 6:**
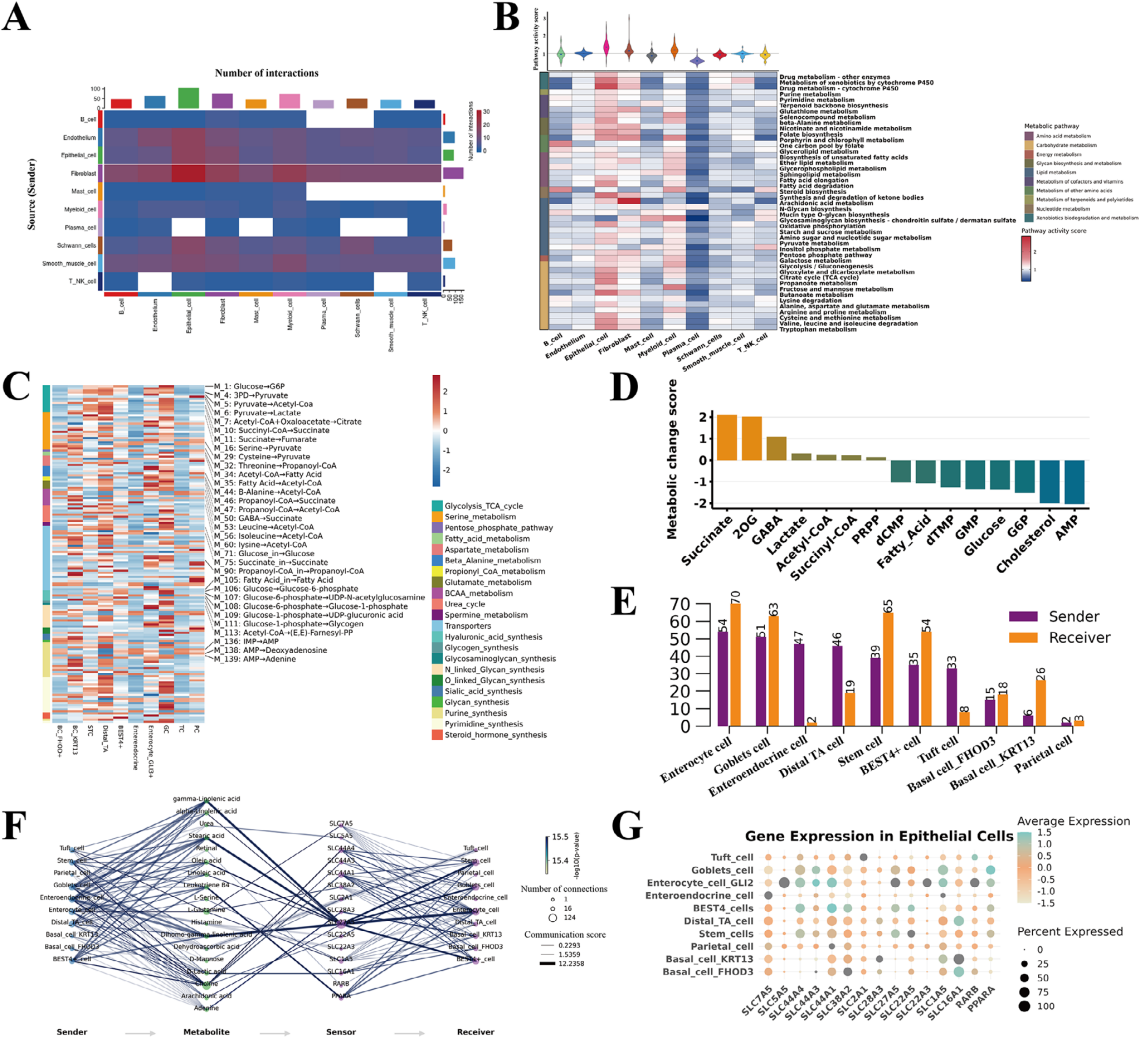
Overview of metabolic characteristics of epithelial cells. A) The heat map shows the number and intensity of interactions between different epithelial subcell types. B) The average pathway activity score of different major cell types (top). Metabolic pathway activities of different major cell types (bottom). C) Profile of the predicted fluxome of metabolic modules. D) The top accumulated and depleted metabolites predicted in epithelial cells. E) Bar plot shows the event number of different epithelial cell types as sender or receiver. F) Visualization of the communication flow from sender metabolite to sensor in receiver. G) The expression of the key sensors in different epithelial cell types.

Intercellular communication plays a pivotal role in coordinating metabolic flexibility and maintaining tissue homeostasis, which is critical for yak high‐altitude hypoxia adaptation. The intercellular communication among different cell types revealed that epithelial cells acted as the major signal receivers, establishing extensive interactions with other cell types, whereas fibroblasts exhibited the highest level of sender signaling. These findings highlighted the central role of epithelial cells in GIT (**Figure** **6**A). Metabolic pathway activity scores using a computational pipeline based on the scRNA‐seq indicated that epithelial cells displayed the highest metabolic activity, followed by fibroblasts and myeloid cells (Figure 6B). Among the 45 identified metabolic pathways (where at least one cell type showed significant enrichment, defined as pathway activity score > 1 and permutation test *p* < 0.01), epithelial cells exhibited elevated activity across most pathways. Notably, butanoate metabolism, TCA cycle, and fatty acid elongation pathways were particularly enriched compared to other cell types. These results indicated that the unique metabolic features of epithelial cells, particularly the enrichment of butanoate metabolism, may synergize with microbial fermentation of high‐fiber diets to efficiently produce butyrate, thereby enhancing epithelial energy homeostasis and barrier integrity to facilitate adaptation to high‐altitude environments.

To further delineate metabolic activities across epithelial cell subtypes, we applied scFEA to infer metabolic fluxes. As shown in Figure 6C, goblet cells showed significantly elevated flux from glucose to glucose‐6‐phosphate (G6P), as well as from G6P to glucose‐1‐phosphate (G1P), and ultimately from G1P to glycogen, indicating that goblet cells not only play a role in immune protection within the GIT but also contribute to optimizing energy supply through glycogen storage, thereby helping yaks adapt to the challenges of high‐altitude hypoxia. Additionally, the flux from pyruvate to lactate was markedly higher in PCs than in other subtypes. Quantitative assessment revealed a substantial accumulation of succinate and 2‐oxoglutarate (2OG) in epithelial cells, whereas key metabolites in glycolysis and the TCA cycle, such as glucose, G6P, and AMP, were significantly depleted (Figure 6D). Furthermore, we found that enterocytes exhibited the strongest characteristics as both signal senders and receivers using the Mebocost framework. Goblet cells and intestinal stem cells also displayed high receptor activity, consistent with their elevated metabolic activity (Figure 6E). Further analysis of metabolite‐sensor communications revealed that unsaturated fatty acids play a prominent role in epithelial metabolic interactions. Notably, members of the *SLC* gene family functioned as key sensors in metabolite‐mediated signaling, with *SLC27A5* being particularly active (Figure 6F). The expression levels of key metabolic sensors across epithelial subtypes aligned with the predicted signal‐receiving strength of each cell type (Figure 6G). These metabolic features may reflect an important adaptive strategy for yak survival in high‐altitude environments, namely optimizing nutrient utilization and maintaining GIT barrier integrity under harsh plateau conditions through enhanced glycolysis and *SLC* gene family‐mediated signaling in coordination with microbial metabolism.

### 2.7 The Pattern of Microorganisms, Metabolites, and Epithelial Cells Involved in Nutrient Absorption and Energy Metabolism in Yaks

**Figure 7:**
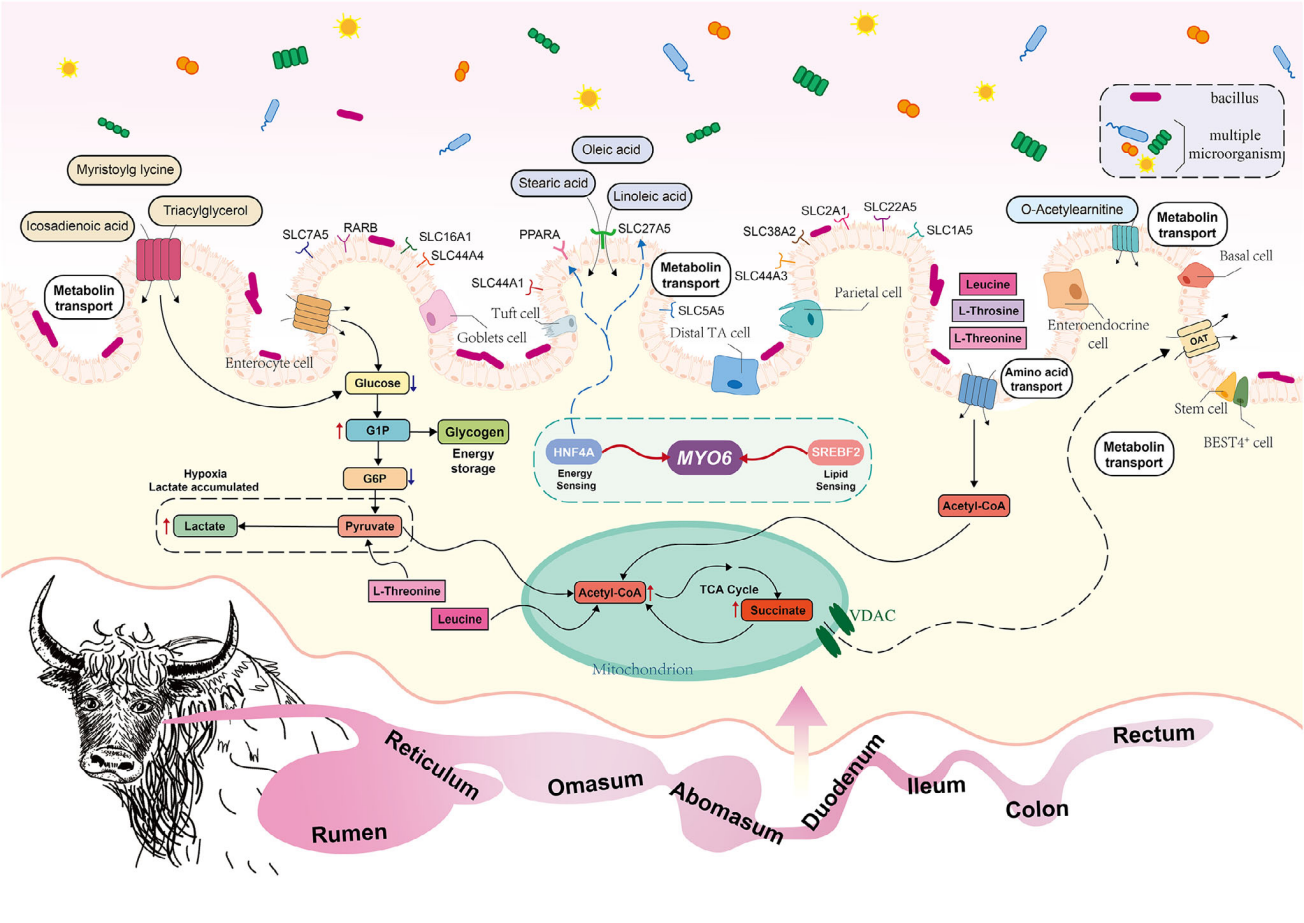
The unique pattern of nutrient absorption and energy metabolism in yak gastrointestinal tract (GIT).

The comprehensive analyses of scRNA‐seq, genomic, microbiome, and metabolome data revealed that epithelial cell types are key contributors to yak production performance, potentially mediating the production of unsaturated fatty acids through highly expressed fatty acid sensors such as *SLC27A5* and *PPARα*, and interacting with cell‐type and spatially specific colonization of *Bacillus* to promote efficient lipid metabolism and immune regulation in the yak GIT, thereby enabling adaptation to the challenges of high‐altitude hypoxic and cold conditions. Different epithelial subtypes exhibited divergent energy metabolic strategies, highlighting the unique adaptive features of yaks. For instance, GCs enhanced glycogen synthesis to support energy storage under high energy demand conditions, whereas PCs showed elevated lactate production and glycolytic flux, suggesting anaerobic ATP generation under stress conditions. In addition, the yak‐specific expression pattern of *MYO6*, regulated by *HNF4A* and *SREBF2*, indicated a potential role in regulating lipid transport and membrane stability, thereby facilitating energy and nutrient uptake by epithelial cells. These results demonstrated that the unique host–microbe–metabolite interactions and transcriptional regulation in the yak GIT confer flexible and efficient energy supply strategies to meet adaptive adjustments and high energy demands in high‐altitude cold environments (**Figure** **7**).

## 3 Discussion

In this study, we generated the first comprehensive multi‐tissue single‐cell transcriptomic atlas of the yak GIT, encompassing 117019 high‐quality cells and 54 distinct cell subtypes. Cross‐species and cross‐tissue comparative analyses identified *HNF4A* and *SREBF2* as GIT‐specific transcription factors targeting the key gene *MYO6*, revealing unique transcriptional patterns with implications for nutrient absorption and energy metabolism under high‐altitude hypoxia conditions in yaks. Integrating this resource with population genetics data revealed that epithelial cells exhibit the strongest association with body weight among all cell types in yaks, with *SLC27A5* and *PPARA* serving as key receptors mediating intercellular communication. In addition to characterizing the microbial and metabolic features along the GIT, this work identified the spatial colonization pattern of Bacillus, which, through interactions with epithelial metabolic activities, influenced the accumulation of succinate, butyrate, and glycogen, thereby enhancing glycolysis and the TCA cycle to improve the flexibility of energy supply under hypoxic conditions. These results suggest that host–microbe–metabolite crosstalk represents a critical mechanism by which yaks optimize nutrient utilization, energy metabolism, and barrier integrity in extreme high‐altitude environments. Collectively, these findings provide novel molecular insights into yak adaptation to hypoxic environments at high altitudes and constitute a valuable resource for advancing our understanding of high‐altitude adaptation.

The adaptability of the yak GIT is one of the major manifestations of high‐altitude adaptation, and its unique features in nutrient absorption and energy utilization are crucial for survival and reproduction under hypoxic conditions at high altitudes. In this study, the discovery of the rare BEST4+ cell type, which was absent in cattle,^[^ ^19^ ^]^ donkeys,^[^ ^20^ ^]^ and buffalo^[^ ^31^ ^]^ living at low altitudes, suggests its important role in high‐altitude adaptation. Specifically, these cells may interact with enteroendocrine cells and secrete electrolytes to promote mucus layer formation, thereby enhancing nutrient absorption efficiency and maintaining barrier integrity. Among all major cell types, immune cells accounted for a large proportion, with notable expression of *IFNG* in T cells. *HIF1α* has been demonstrated as a genuine metabolic regulator of *IFNG* induction in hypoxic T cells, influencing cell growth, activation, and proliferation driven by activation through the promotion of glycolysis.^[^ ^32^ ^]^ These findings indicate that the yak GIT enhances immune defense to cope with the challenges of nutrient absorption under extreme environments, thereby sustaining growth and development in high‐altitude hypoxia. Furthermore, *HNF4A* and *SREBF2* exhibited strong tissue specificity in the yak FC and intestine, maintaining metabolic homeostasis and cellular function by regulating acetyl‐CoA metabolism‐related genes and cholesterol regulation, respectively.^[^ ^33^, ^34^ ^]^ Their target gene *MYO6* was significantly upregulated in yak epithelial cells. As the only minus‐end‐directed actin motor, *MYO6* plays a critical role in intracellular transport and the maintenance of epithelial barrier homeostasis.^[^ ^35^ ^]^ These results suggest that the *HNF4A*/*SREBF2*‐*MYO6* axis may be an important regulator of production performance under hypoxic high‐altitude conditions and serve as a key molecular target for improving digestion and nutrient absorption efficiency in yaks. In addition, we identified a unique association between yak GIT epithelial cells and body weight trait, which is consistent with previous studies showing that the functions of specific cell types are conserved across mammalian species.^[^ ^36^, ^37^, ^38^ ^]^ Highly heritable genes, including *NFATC1*, *COX8A*, and *HIF1A*, were found to be associated with body weight, highlighting them as candidate genes underlying the unique high‐altitude hypoxia adaptation of yaks.

The adaptive regulation of gut microbiota and host metabolism is crucial for yak survival under high‐altitude hypoxia conditions. The dynamic changes and characteristics of F/B ratio in the GIT microbiota indicate that, compared with low‐altitude animals, yak microbial communities have undergone long‐term selection in high‐altitude hypoxic environments, enabling the utilization of more GIT sites to digest fibrous forage in response to plateau ecological pressures. Moreover, yaks achieve efficient fiber digestion through coordinated multichamber fermentation in the stomach while enhancing nutrient absorption in the SI, thereby maximizing energy acquisition under the cold and hypoxic conditions of the QTP. Notably, *Bacillus* species, due to their broad adaptive features, exhibit high bioavailability.^[^ ^39^ ^]^ Their distribution within yak GIT epithelial and immune cells suggested a role in maintaining intestinal immune homeostasis through the secretion of antimicrobial substances, while producing multiple hydrolases that significantly improve the digestibility of fibrous forage, thus providing essential support for energy utilization and immune adaptation under harsh plateau conditions. We also observed elevated levels of lipid‐ and amino acid–related metabolites in the yak GIT. These metabolites participate in the TG/fatty acid cycle, which is essential for thermogenesis, and contribute to resisting oxidative stress in hypoxia through high deamination rates and the production of glutathione precursors.^[^ ^40^, ^41^ ^]^ These findings demonstrate the flexible regulation of energy expenditure in the yak GIT, which is vital for growth and development in cold and hypoxic environments. In addition, epithelial cells showed important contributions to high‐altitude adaptation in yaks. The accumulation of key metabolites such as butyrate, succinate, lactate, and 2OG highlights unique mechanisms for rapid responses to high‐altitude hypoxia and nutritional stress, including preferential glycogen storage to sustain the mucus barrier and reliance on anaerobic glycolysis for rapid energy supply. Furthermore, fatty acids interact with specific sensors such as *SLC27A5* to trigger intracellular signaling that coordinates metabolic states. *SLC27A5* is essential for long chain fatty acid transport and bile acid metabolism^[^ ^42^ ^]^ and is regulated by *HNF4A*.^[^ ^43^ ^]^ We proposed that *HNF4A*, by modulating *SLC27A5*‐mediated lipid metabolism, may play a key role in the adaptation of the yak intestine to extreme high‐altitude environments.

The unique GIT cellular atlas and microbial‐metabolic features of yaks provide novel molecular insights into their adaptation to high‐altitude hypoxic environments. However, this study does have certain limitations. First, the number of individuals analyzed was limited, and the environmental contexts considered should be further expanded, including developmental stages and dietary variations. Moreover, future work should integrate single‐cell omics data from a broader range of cattle breeds and increase genomic sampling to improve the accuracy of cell‐type annotation and elucidate the impact of genetic variation. Second, the functions of the key genes and microorganisms identified in this study require further validation with larger sample sizes.

## 4 Experimental Section

### Ethics Statement, Sampling, and Sequencing

All the animal experiments and procedures in this study were approved by the Lanzhou Institute of Husbandry and Pharmaceutical Sciences of the Chinese Academy of Agricultural Sciences under the grant number LIHPS‐20230825 (Table S1, Supporting Information). The methods were implemented strictly in accordance with the guidelines of this institute. Considering adult yaks possess relatively stable microbial communities, six yaks were selected, six 3.5‐year‐old yaks (three males and three females) were collected in October from Gannan Tibetan Autonomous Prefecture, Gansu, China. All yaks were raised under a traditional transhumance system, which is the most common and characteristic feeding strategy on the plateau, thereby better reflecting the GIT features of yaks under high‐altitude hypoxia conditions. All the collected yaks were humanely euthanized after undergoing a 12‐h fast. To greatly minimize the potential contamination across the GIT regions, animal carcasses were laid on the sterilized table in their natural way without unnecessary moving for sampling. Sterile rope was used to tie off the GIT regions separately, whenever possible, all the gastrointestinal content samples (≈10–30 mL) were taken immediately following dissecting tissues, and then were filtered with four layers of sterile medical gauze. For the scRNA‐seq, eight types of tissues from three yaks (one male and two females), including rumen (*n* = 3), reticulum (*n* = 1), omasum (*n* = 2), abomasum (*n* = 2), duodenum (*n* = 1), ileum (*n* = 2), colon (*n* = 1), and rectum (*n* = 2), were freshly harvested from slaughtered samples. For the metagenomics and metabolomics studies, 50 gastrointestinal content samples of nine sites along the GIT including stomach (rumen, reticulum, omasum, and abomasum), small intestine (duodenum, jejunum, and ileum), and large intestine (cecum and colon), and rectum fecal samples were collected from five yaks (two males and three females, see Supporting Information).

### Raw scRNA‐seq Data Processing

Illumina bc12fastq software was performed to convert raw sequencing data to FASTQ formant, then the FASTQ files were processed and aligned to the yak reference genome *BosGru3.1* (RefSeq assembly accession GCA_0 058 87515.3) using the “mkref” function of Cell Ranger software (version 7.0.1) from 10× Genomics, with unique molecular identifier (UMI) counts summarized for each barcode. The UMI count matrix was then analyzed using Seurat (version 4.0.0) R package.^[^ ^44^ ^]^ A mean number of 8359 cells per sample (range: 5828–12 189) were obtained. A mean fraction of reads mapped confidently to the genome of 86.76% ± 0.52% (mean ± SD), a mean number of 348 307 150 ± 27 327 115 reads for each sample library, and a mean sequencing saturation of 53.25% ± 18.04% (Table S2, Supporting Information). Then, a set of criteria were conducted to remove low‐quality cells and likely multiple captures (see Supporting Information, Table S2, Supporting Information).

### Cell Clusters Identification and Annotation

The “merge” function was used tto merge the preprocessed data sets from same tissues separately. Top 2000 highly variable genes (HVGs) were calculated using the function “FindVariableGenes” of Seurat package (selection.method = vst, nfeatures = 2000). Harmony (version 0.1.1)^[^ ^45^ ^]^ method was performed to reduce three batch effects, including source, method and individuals (Figure S1, Supporting Information). Dimensionality reduction plots before and after Harmony correction were provided in Figure S1 (Supporting Information), showing that batch effects were effectively mitigated. Graph‐based clustering was performed to cluster cells according to their gene expression profile with the “FindClusters” function (resolution = 0.4). Cells were visualized using a UMAP algorithm with the “RunUMAP” function and t‐SNE with the “RuntSNE” function, respectively. Each cell cluster was annotated artificially based on the marker genes that has been reported in the relevant scientific literature.

The “FindAllMarkers” function and “FindMarkers” function with default parameters (|log2FoldChange (FC)| ≥ 0.25 and adjusted *P_adj*‐value ≤ 0.05) were performed to identify the differential analysis of genes in cell clusters and statistical significance was determined using the Wilcoxon rank‐sum test. Each cell type was annotated by integrating data from the online PanglaoDB database (), known classical markers from extensive published literature, and functional enrichment analyses of the top 100 highly expressed genes. This comprehensive approach significantly enhanced the accuracy of cell‐type annotation results.

### Cell‐Cycle Index Estimation

The cell‐cycle index analysis was performed according to the method with the AUCell scoring to the cycling and noncycling gene sets of each cell type.^[^ ^46^ ^]^ In general, the greater the logarithm ratio of the number of cycling per cell type to the number of non‐cycling cells, the stronger the ability of cells to divide rapidly.

### Transcription Factor, Regulons Modules, and Single‐Cell Regulatory Network Analysis

The pySCENIC^[^ ^47^ ^]^ (version 0.12.1) package in Python was used to inferred the regulatory network with the default parameters. The Homologene package (version 1.4.68.19.3.27) in R was utilized to one‐to‐one convert homologous genes of humans and yaks which row counts derived from the Seurat object, a total of 16203 homologous genes were retained for subsequent analysis. The TFs analysis was performed including specific TFs identification, genes directly targeted by the TFs prediction, the regulon activity score and specificity score calculation, the regulon modules using the connection specificity index identification, and the different regulon modules determination (see Supporting Information).

### High‐Dimensional Weighted Gene Co‐Expression Network Analysis

The characteristic gene modules of different tissues were identified by the hdWGCNA R package.^[^ ^48^ ^]^ The detailed process involves selecting genes that are expressed in at least 5% of the cells to create Weighted Gene Co‐expression Network Analysis (WGCNA) objects. The “MetacellsByGroups” function is employed to construct “metacells”, which helps maintain cell heterogeneity while reducing sparsity. The “TestSoftPowers” function is utilized to calculate the optimal soft power threshold for constructing a co‐expression network, with the optimal threshold determined in this study being 7. Subsequently, the “ModuleEigengenes” function is applied to compute the module feature genes. A co‐expression network is constructed using the “ConstructNetwork” function, and the correlation of feature genes is calculated using the “ModuleConnectivity” function. Finally, UMAP is used to embed the co‐expression network topology overlap matrix (TOM) into a two‐dimensional manifold.

### Cross‐Species Multi‐Tissue Cell Landscapes Analysis

The high‐quality data of six tissues of dairy cow that includes the rumen, reticulum, omasum, abomasum, ileum, and rectum from public databases were downloaded for cross‐species multi‐tissue cell landscapes analysis (GSE176512).^[^ ^19^ ^]^ All data were processed according to the parameters outlined in the “Raw scRNA‐sequencing data processing” section. After acquiring high‐quality data, the Ensembl Biomart tool was employed to convert the gene names of yaks and cows into their corresponding human orthologous genes for subsequent analysis. Subsequently, the reciprocal principal component analysis (RPCA) method was utilized to integrate the cross‐species scRNA‐seq data from both species. Upon securing high‐quality data, the BioMart R package was used to convert the gene names of yaks and cows. All cross‐species analyses at the single‐cell level were conducted using 19316 one‐to‐one homologous genes between yaks and cows. The cell‐type correlation between yaks and cows were determined by Spearman's correlation coefficient using the calculated average expression values of the top 2000 highly variable genes in each cell type.

### Transcriptional Similarity Analysis between Cross‐Species Cell Types

MetaNeighbor software (v1.20.0) is a powerful and accurate tool for evaluating the repeatability of cell types both within and between species.^[^ ^49^ ^]^ The analysis process, as detailed in the documentation (), primarily involves converting single‐cell transcriptome data into the SingleCellExperiment format, constructing a gene expression matrix, and utilizing highly variable genes to calculate AUROC scores. Finally, hierarchical clustering is performed based on these AUROC scores to illustrate the relationships between cell types across species and within species.

### Genome‐Wide Association Study (GWAS) Analysis

A total of 247 yaks whole‐genome sequencing data (an average sequencing depth of 8.8X per samples) were collected and analyzed with body weight traits. All raw reads were mapped to the yak reference genome BosGru3.1 (RefSeq assembly accession GCA_0 058 87515.3) to detect the mutation sites of each sample. The low‐quality SNPs with “Minor Allele Frequency (MAF) < 0.05, Hardy–Weinberg Equilibrium (HWE) test result p < 1e6, and Dosage R‐Squared (DR2) < 0.9” were removed, then 12411513 SNPs that related to BW traits were obtained for subsequent analysis. The Fixed and Random Model Circulating Probability Unification (FarmCPU) algorithm in the rMVP R package was performed to identify and visualize the GWAS results.^[^ ^50^ ^]^

### Enrichment Analysis Between Cell Types and Complex Traits

The pathway‐based polygenic regression method (scPagwas) employs a linear regression of GWAS signals derived from the pathway activation of transformed scRNA‐seq data. This approach identifies a set of trait‐related genes, which are subsequently utilized to infer the most trait‐related cell types. Therefore, the scPagwas software (version 2.0.0) was performed the enrichment analysis to explore the correlations between complex traits and cell types.^[^ ^51^ ^]^ To enhance the accuracy and comprehensiveness of the analysis results, this work primarily removed duplicates and converted homologous genes between yaks and humans. The human KEGG pathways from the KEGG database () was downloaded for next analysis. The LD values of SNPs in the GWAS dataset were calculated, and SNPs with LD greater than 0.8 were retained. Ultimately, 81 402 SNPs annotated to 7086 genes were used as the input GWAS data for subsequent analyses. The singular value decomposition (SVD) method was used to calculate the SVD value between single cell and cell types by “Pathway_pcascore_run” function in scPagwas software. By performing linear regression, scPagwas prioritizes a set of trait‐relevant genes and uncovers cell subpopulations pertinent to the trait under investigation, including assessing pathway activity for each cell to obtain pathway scores, incorporating GWAS summary data into the analysis pipeline, associating SNPs from GWAS data with corresponding genes, annotating pathways and SNPs to establish their relationships, associating pathway blocks with PCA scores to evaluate the relationship between pathway activity and the trait, conducting regression analyses for each cell type to identify trait‐relevant cell subpopulations, developing scPagwas scores based on regression results to assess each cell's association with the trait, calculating Pearson Correlation Coefficients for each gene to identify genes associated with the trait, computing TRS for the top 1000 genes to evaluate their activity within cells, and adjusting TRS scores for single cells to enhance result reliability. Finally, the Fisher's test and Chi‐square test within each cell type were utilized to perform enrichment analysis between trait‐relevant genes and active pathway genes.

### Metabolic Activity Evaluation

The metabolic pathway activity of different cell types was calculated using the widely employed single‐cell metabolic landscape pipeline ().^[^ ^52^ ^]^ The relative expression level was determined by calculating the average expression level of metabolic genes in each cell type and comparing it with the average expression level across all cell types. Each metabolic pathway is defined by the sum of the weighted average relative expression levels of the constituent genes. Furthermore, genes exhibiting non‐zero relative expression levels in each pathway and expressed in at least 25% of cells were selected for analysis to enhance the accuracy of pathway activity detection. Finally, the cell‐type labels were randomly rearranged 5000 times and compared with the pathway activity scores from the original, non‐rearranged dataset to evaluate the statistical significance of pathway activity in specific cell types.

Single‐cell Flux Estimation Analysis (scFEA) is a method specifically designed to analyze the metabolic networks of single cells, revealing their metabolic activities by inferring metabolic fluxes.^[^ ^53^ ^]^ Initially, the yak gene names were converted to their human orthologs, and subsequently, the metabolic flux and abundance for each cell type was calculated using “scFEA.py”.

### Function Enrichment Analysis

The online g:Profiler () was used to perform GO and KEGG function enrichment analysis for gene sets.^[^ ^54^ ^]^ All the terms and pathways were considered statistically significant with *p*‐value < 0.05.

### Metabolome Analysis

The raw data collected using MassLynx (V4.2) is processed by Progenesis QI software for peak extraction, peak alignment and other data processing operations, based on the Progenesis QI software online METLIN database and self‐built library for identification, and at the same time, theoretical fragment identification and mass deviation All are within 100 ppm.

After normalizing the original peak area information with the total peak area, the follow‐up analysis was performed. Principal component analysis (PCA) and Spearman correlation analysis were used to judge the repeatability of the samples within group and the quality control samples. The identified compounds are searched for classification and pathway information in KEGG, HMDB and lipid maps databases. According to the grouping information, calculate and compare the difference multiples, *T* test was used to calculate the difference significance p‐value of each compound. The R language package ropls was used to perform OPLS‐DA modeling, and 200 times permutation tests were performed to verify the reliability of the model. The VIP value of the model was calculated using multiple cross‐validation. The screening criteria are FC > 1, *p*‐value < 0.05 and VIP > 1. The different metabolites of KEGG pathway enrichment significance were calculated using hypergeometric distribution test.

The R package Prcomp (v3.6.1) was performed to conduct PCA with the parameters “scale: uv scaling”.

### Weighted Correlation Network Analysis of the Metabolites

All the detected metabolites were used to construct the co‐expression network using the WGCNA R package (v1.73).^[^ ^55^ ^]^ The Wilcoxon rank sum was performed to evaluate the associations between module eigengenes and tissues. The metabolites with module membership″ >0.9 and “metabolite significance” >0.9 were considered as the key metabolites in the modules.

### Metagenome Assembly and Binning

Firstly, the fastp software (v 0.23.4) with the parameters “‐W 50 ||‐M 20 ‐5 ‐n 0‐g ‐A ‐D” was used to obtain the clean reads from the raw reads, including trimming adapter sequences and low‐quality sequences.^[^ ^56^ ^]^ Then the Bowtie2 software (v 2.5.1) was performed to map the clean reads to the yak reference genome *BosGru3.1* (RefSeq assembly accession GCA_0 058 87515.3) for decreasing potential DNA contamination from the host.^[^ ^57^ ^]^ The remaining paired reads were used for the subsequent analysis.

The Megahit software (v1.2.9) is considered as an efficient assembly software.^[^ ^58^ ^]^ To improve the assembly results of less abundant species in the samples, Megahit software was used to assemble each sample and co‐assemble samples by tissue grouping, and filtered contig sequences shorter than 300 bp. The assembly results of each sample and group were evaluated using QUAST software (v 0.0.14).^[^ ^59^ ^]^ The MetaBAT2 software with the default parameters was used to performed the metagenomics binning.^[^ ^60^ ^]^ The completeness and contamination of all bins that were dereplicated using dRep software^[^ ^61^ ^]^ were detected by CheckM.^[^ ^31^ ^]^

### Taxonomic Assignments and Functional Annotation

The 533 MAGs (completeness ≥ 70% and contamination ≤ 10%) were obtained to perform the taxonomic using “classify_wf” workflow in the GTDB‐Tk (v 1.2.0) software.^[^ ^62^ ^]^ The Prodigal (v 2.6.3) software was conducted to identify the coding region in the genome with “meta” model.^[^ ^63^ ^]^ The “run_dbcan.py” script in the program dbCAN2 (v 3.0.2)^[^ ^64^ ^]^ and the “emapper.py” script in the program eggnog‐mapper (v 2.1.12) were performed to match protein sequences to entries of the CAZy database and the gene catalogs to obtain the CAZymes results and specific COGs results. Additionally, the functional assignments of protein sequences were aligned against the KEGG protein database by the best hit with the criteria of *E* value < 1e‐5.

The abundance profiles of genes were normalized by the transcripts per million (TPM) that correspond to reads in this study using Salmon software. The abundances of annotated genes were used to calculate the relative abundances of taxa, COGs and CAZymes. The phylum, genus, and species relative abundances were also calculated by the relative abundances of genes.

### Microbial Identification Using the SAHMI Pipeline

Single‐cell Analysis of Host‐Microbiome Interactions (SAHMI) is an analytical pipeline () designed to identify microbial abundance in single‐cell transcriptomes.^[^ ^65^ ^]^ This pipeline effectively removes contaminants and false positives from scRNA‐seq data of mammalian host tissues, thereby providing a more comprehensive understanding of microbial infection in host cells. The analysis proceeds through several steps: First, reads are compared against a comprehensive database using Kraken2, and classified with Kraken2Uniq. Subsequently, a k‐mer correlation test is conducted to evaluate the quantitative relationship between the total k‐mers and the unique k‐mers of each microbial taxon, utilizing Spearman correlation analysis (with *p*‐value < 0.05 indicating statistical significance). Finally, a correlation matrix of microbial UMIs and barcodes is constructed, filtering out contaminants and false positives to facilitate host‐microorganism interaction analysis.

### Cell–Cell Interaction Analysis

Cellular communication patterns between different cell types were implemented using the CellChat (version 1.6.1) R package based on the label‐based mode with default parameters, which is a database containing literature‐supported ligand‐receptor interactions in mice and humans.^[^ ^66^ ^]^ Yak gene symbols were mapped to human orthologs for the subsequent cellular communication analysis. The interactions of all the annotated epithelial cell types were assessed for the comprehensive dynamics of cellular communication networks by calculating the average intensity. The ligand‐receptor pairs with *p*‐value < 0.05 were considered to be significant.

Metabolic cell–cell communication analysis was performed by MEBOCOST, which is a Python‐based tool that inferred metabolite‐mediated cell communication from single cell.^[^ ^67^ ^]^ Briefly, the enzyme‐sensor co‐expression score of metabolite enzyme and sensor pairs were calculated, and then normalized by the mean background co‐expression score obtained from the random shuffling procedure.

### Hematoxylin and Eosin (H&E) Staining and Immunostaining

Tissues were fixed in 4% paraformaldehyde at 4 °C overnight, embedded in paraffin, and sectioned at 5 µm. For H&E staining, standard hematoxylin and eosin protocols were applied for morphological assessment. For IHC, sections underwent antigen retrieval in sodium citrate buffer (96 °C, 10 min), blocked with 5% BSA in TBST, treated with 3% H_2_O_2_, incubated with primary antibodies overnight at 4 °C, and with secondary antibodies for 1 h at room temperature. Detection was performed using DAB substrate, followed by hematoxylin counterstaining. For IF, similar steps were followed without H_2_O_2_ treatment, using fluorescent secondary antibodies and DAPI for nuclear staining. Images were captured using an Olympus BX51 microscope.

### Statistical and Reproducibility

No statistical method was used to predetermine the sample size. All the statistical analyses and data visualization were performed in the R environment if not specified and all analyses were not randomized for ensuring maximum reproducibility. The following were considered as statistical significance: ^*^ *p* < 0.05, ^**^ *p* < 0.01, and ^***^ *p* < 0.001.

## Conflict of Interest

The authors declare no conflict of interest.

## Author Contributions

C.H., C.L., and P.Y. worked on conceptualization. C.H., M.Z., Q.Z., and Q.Y. worked on investigation. C.H., M.Z., G.Y., C.L., and P.Y. performed methodology. P.B., M.C., C.L., and P.Y. worked on resources. X.G., C.L., and P.Y. performed supervision. C.H., W.R., X.M., and Y.L. worked on validation. C.H. and P.Y. worked on visualization. C.H. wrote the original draft. C.H., C.L., and P.Y. reviewed and edited the writing.

## Supporting information



Supporting Information

Supplemental Table 1

Supplemental Table 2

Supplemental Table 3

Supplemental Table 4

Supplemental Table 5

Supplemental Table 6

Supplemental Table 7

Supplemental Table 8

Supplemental Table 9

Supplemental Table 10

Supplemental Table 11

Supplemental Table 12

Supplemental Table 13

## Data Availability

The data that support the findings of this study are available in the supplementary material of this article.
