# Supplementary material for: The High‐Altitude Adaptation Characteristics of Microbiota‐Host Cross‐Talk in Yak Gastrointestinal Track
Source: Adv Sci (Weinh). 2025 Oct 27;13(1):e14862. doi: 10.1002/advs.202514862 (PMC12767066; doi:10.1002/advs.202514862)
Supplement: Supplementary file 1 — Supporting Information [file ADVS-13-e14862-s004.docx]

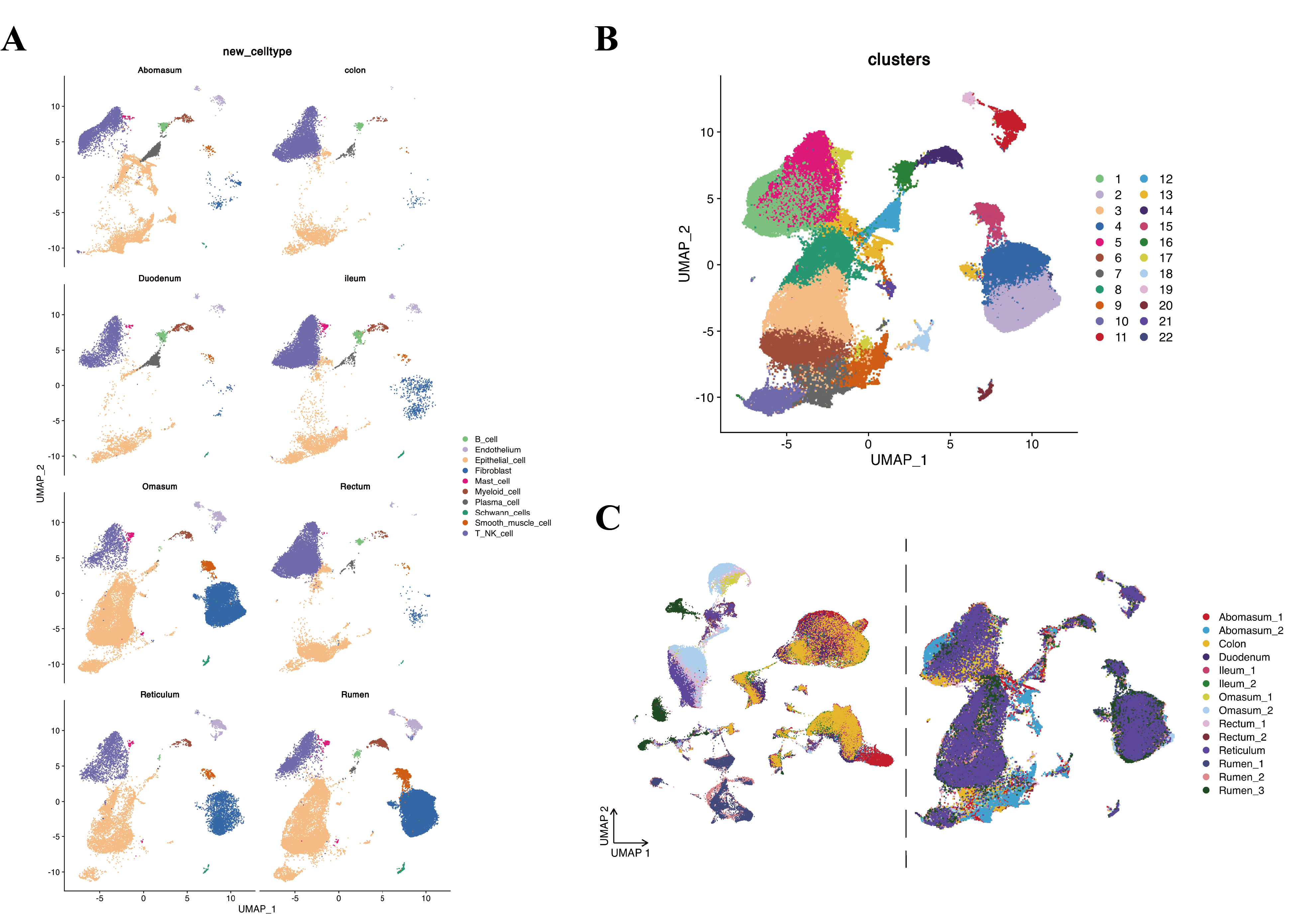


**Supplementary Figure S1.** Details of scRNA-seq data. (A) The major cell types annotations of different tissues. (B) Details of cluster results. (C) Distributions of different samples before (*left*) and after (*right*) Harmony integration.


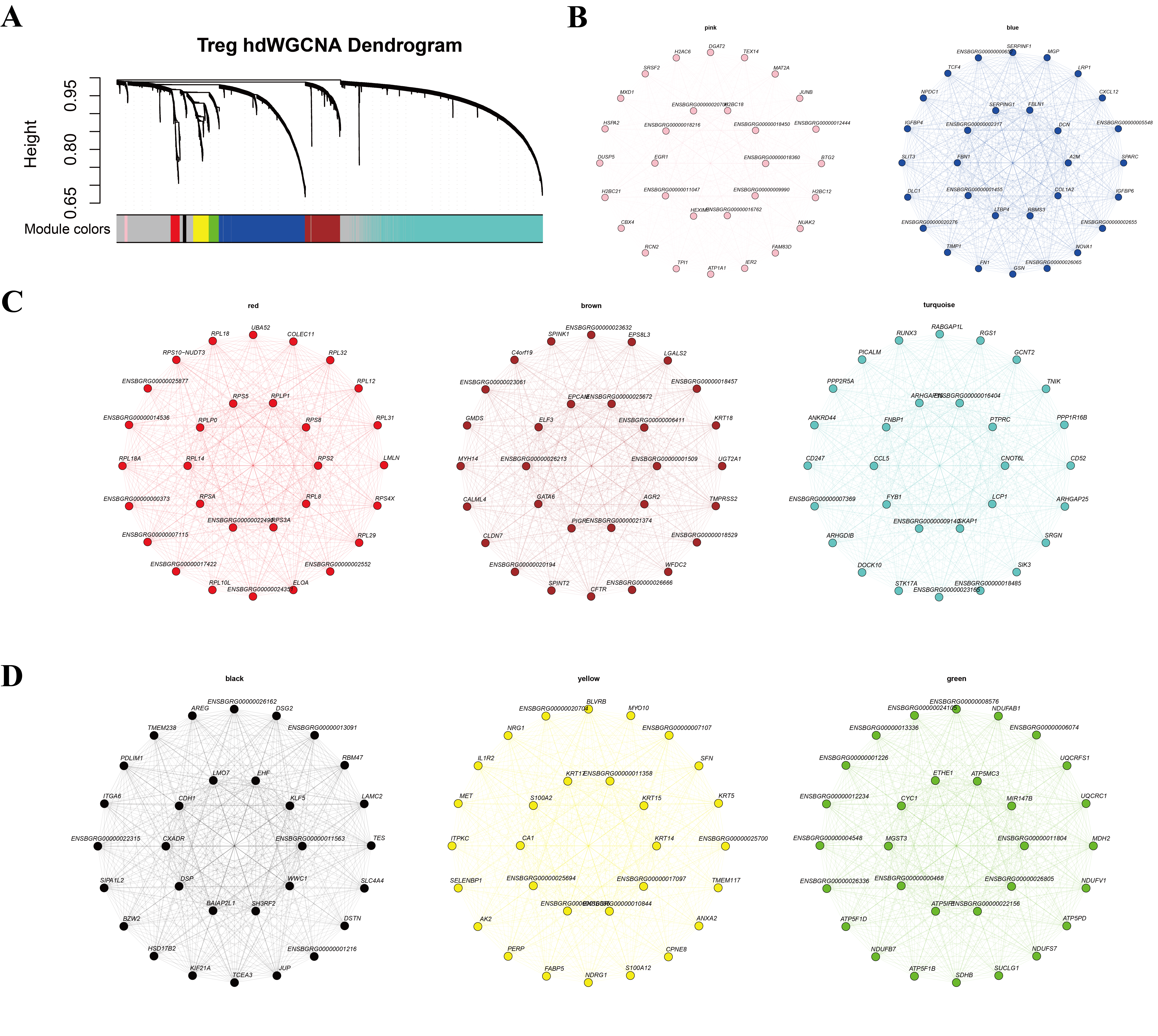


**Supplementary Figure S2.** Co-expression network analysis of different tissues in yak GIT. (A) Visualize the 8 modules in a scale-free network using a dendrogram. (B-D) Co-expression plots for 8 modules.


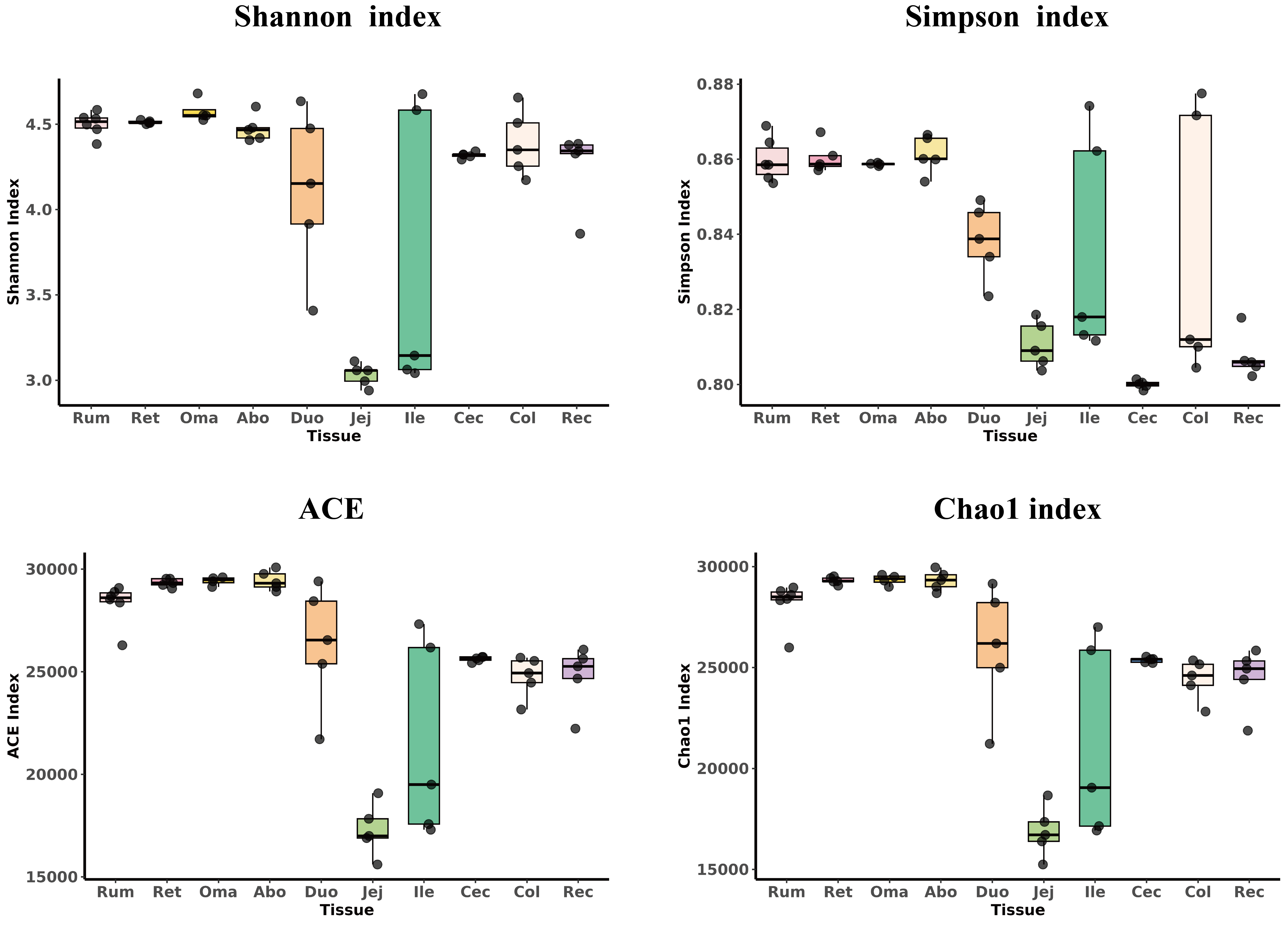


**Supplementary Figure S3.** Results of alpha diversity analysis.
